# Heat Shock Protein Member 8 Is an Attachment Factor for Infectious Bronchitis Virus

**DOI:** 10.3389/fmicb.2020.01630

**Published:** 2020-07-10

**Authors:** Pengpeng Zhu, Chenfei Lv, Chengxiu Fang, Xing Peng, Hao Sheng, Peng Xiao, Nishant Kumar Ojha, Yan Yan, Min Liao, Jiyong Zhou

**Affiliations:** Key Laboratory of Animal Virology of Ministry of Agriculture, Zhejiang University, Hangzhou, China

**Keywords:** infectious bronchitis virus, HSPA8, virus entry, receptor binding domain, attachment factor

## Abstract

Although infectious bronchitis virus (IBV) is the first coronavirus identified, little is known about which membrane protein of host cells could interact with IBV spike protein and facilitate the infection by the virus. In this study, by using a monoclonal antibody to the S1 protein of IBV M41 strain, we found that heat shock protein member 8 (HSPA8) could interact with spike protein of IBV. HSPA8 was found to be present on the cell membrane and chicken tissues, with highest expression level in the kidney. Results of co-IP and GST-pull-down assays indicated that the receptor binding domain (RBD) of IBV M41 could interact with HSPA8. The results of binding blocking assay and infection inhibition assay showed that recombinant protein HSPA8 and antibody to HSPA8 could inhibit IBV M41 infection of chicken embryonic kidney (CEK) cells. Further, we found that HSPA8 interacted with the N-terminal 19–272 amino acids of S1 of IBV Beaudette, H120 and QX strains and HSPA8 from human and pig also interacted with IBV M41-RBD. Finally the results of binding blocking assay and infection inhibition assay showed that recombinant HSPA8 protein and antibody to HSPA8 could inhibit IBV Beaudette strain infection of Vero cells that were treated with heparanase to remove heparan sulfate from the cell surface. Taken together, our results indicate that HSPA8 is a novel host factor involved in IBV infection.

## Introduction

Infectious bronchitis virus (IBV) is a member of genus gamma-coronavirus in the family *Coronaviridae*, causing serious economic losses to the poultry industry (Cavanagh, 2007). IBV has many genotypes and serotypes circulating in poultry farms leading to continuous outbreaks of infectious bronchitis (IB) disease (Bijlenga et al., 2004). IBV is an enveloped virus with a single stranded unsegmented positive sense RNA genome of about 27 kb size. The IBV virion is made up of spike protein (S), membrane protein (M), nucleoprotein (N), envelope protein (E), and genomic RNA (Lai and Cavanagh, 1997). Spike protein determines the IBV tropism and can be cleaved into the two subunits: S1 and S2 by host furin protein (Cavanagh et al., 1992; Casais et al., 2003). The S1 subunit is responsible for binding to the cell surface receptor, while S2 is responsible for fusion of viral envelope and cellular membrane (Luo and Weiss, 1998; Belouzard et al., 2012).

Interaction between virus protein and cellular receptor is the first step for virus infection (Smith and Helenius, 2004). Different coronaviruses use different cell surface molecules to gain entry into the target cells. For instance, Aminopeptidase N (APN) is the receptor for H229E, TGEV, and PDCoV (Delmas et al., 1992; Breslin et al., 2003; Wang and Huang, 2019); MHV utilizes CEACAM1 as receptor (Williams et al., 1991); ACE2 is the receptor for SARS-CoV (Li et al., 2003) and recently emerged SARS-CoV-2 (Wan et al., 2020); DPP4 is the receptor for MERS-CoV (Raj et al., 2013). Meanwhile, besides cellular receptors, other co-factors in the cell membrane may also influence virus binding. For example, glucose-regulated protein 78 (GRP-78) is an important attachment factor for MERS-CoV infection of host cells (Chu et al., 2018). Calcium dependent (C-type) lectins play a role during SARS-CoV and FCoV infection (Belouzard et al., 2012).

Though IBV was the first coronavirus discovered, study of its host cell receptor goes slowly. Previous studies showed that α-2,3 sialic acid and heparan sulfate (HS) were binding molecules for IBV onto the host cells (Winter et al., 2006; Madu et al., 2007). Recently, Wang et al. reported that IBV entered the cells through clathrin-mediated endocytosis (Wang et al., 2019). The N terminal 253 amino acids of the mature S1 subunit of IBV-M41 (aa 19–272 of the S1 precursor) has been identified as a receptor binding domain (RBD) (Promkuntod et al., 2014). However, the functional receptor for IBV infection is still unknown. In this study, we attempt to identify molecules on host cell membrane responsible for IBV infection. Using immunoprecipitation assay and mass spectrometry analysis, we identified heat shock protein member 8 (HSPA8) as a host cell molecule involved in IBV infection. HSPA8 is a member of HSP70 family and is also referred to as HSP71. It is a conserved protein among different species during evolution (Stricher et al., 2013). A large number of cellular functions have been attributed to HSPA8 (Liu et al., 2012). Previous research showed that HSPA8 can exist in cell membrane and has a role in binding to cell surface by several viruses such as Rotavirus and Human T-Cell Lymphotropic Virus Type 1 (Sagara et al., 1998; Guerrero and Moreno, 2012). Our results suggest that HSPA8 plays a role during initial stages of IBV infection.

## Materials and Methods

### Cells, Viruses, Antibodies, Reagents, SPF Embryonated Eggs, and Chickens

Chicken embryo kidney (CEK) cells were prepared from 17 to 19 day-old SPF embryonated chicken eggs and maintained in DMEM supplemented with 10% FBS at 37°C in 5% CO_2_ atmosphere. Vero and 293T cells were maintained in DMEM supplemented with 10% FBS at 37°C in 5% CO_2_ atmosphere. IBV M41 (GenBank: MK937830.1), H120 (GenBank: MK937831.1) and QX vaccine strains (GenBank: KY933090.1) were purchased from China Institute of Veterinary Drug Control and stored in our laboratory. IBV Beaudette strain was a gift from Prof. DingXiang Liu from South China Agricultural University, Guangdong, P.R. of China. Culture of IBV virus was carried out in bio-safety cabinet (SG603A, BAKER) in bio-safety level 2 plus (BSL2 +) laboratory.

In-house mAbs against IBV S (1H1), M (2B3) and N (4C1) proteins were produced by our research group by the method described previously (Hu et al., 2007). mAb against HSPA8 (ab19136, mouse) and Actin (ab179467, rabbit) were purchased from Abcam. Rabbit IgG (A7016) and mouse IgG (A7028) were purchased from the Beyotime Institute of Biotechnology. mAbs against HSP90AB1 (EM21103, mouse), GAPDH (EM1101, rabbit), GST (EM80701, mouse), GFP (ET1602-7, rabbit), His (R1207-2, mouse), Histone3 (M1306-4, mouse) and polyclonal antibody against HSPA8 (R1511-6, rabbit) were purchased from Huaan Biological Technology. mAb against Flag was purchased from Sigma-Aldrich (F1804, mouse). The secondary antibodies (horseradish peroxidase [HRP]-labeled anti-mouse or anti-rabbit IgG) used for Western blotting were purchased from KPL (Millford, MA). NP-40 lysis buffer (P0013F) was purchased from the Beyotime Institute of Biotechnology. Glutathione-Sepharose beads were purchased from Thermo Fisher Scientific. Ni-NTA agarose (30210) was purchased from QIAGEN. Heparanase I (S31310) was purchased from the Shanghai Yuanye Biological Technology. Specific pathogen free (SPF) chicken embryonated eggs and one-day-old chickens were purchased from Ningbo Chunpai Agricultural Technology Company. The use of chicken tissues in this study was approved by the Committee on the Ethics of Animal of Zhejiang University (ZJU20170388).

### Plasmid Construction and Transfection

HSPA8 gene fragments of different species were amplified from genomic DNA of CEK (chicken), 293T (human) and PK15 (pig) cells by PCR with primers Chicken-HSPA8-F/R, Human-HSPA8-F/R and Pig-HSPA8-F/R listed in Table 1. The fragments were then cloned into the pCMV-flag-N vector between restriction enzyme *EcoR*I and *Kpn*I sites by homologous recombination. M41-S1-RBD (253), Beaudette-S1-253, H120-S1-253, QX-S1-253 gene fragments (the N-terminal 19–272 amino acids of the S1 subunit) were amplified from total RNA extracted from individual specific virus strain infected cells or infected chicken embryo allantoic fluid by RT-PCR with primers M41-S1-RBD(253)-F/R, Beaudette-S1-253-F/R, H120-S1-253-F/R, and QX-S1-253-F/R listed in Table 1. The gene fragments were cloned into pEGFP-C3 vector between restriction enzyme *EcoR*I and *Bam*HI sites by homologous recombination.

**TABLE 1:** Primers used for gene amplification in this study.

Primers	Sequence (5′–3′)
Chicken-HSPA8-F	tggccatggaggcccgaattcGGATGTCAAAGGGACCAGCTGTTG
Chicken-HSPA8-R	gatccccgcggccgcggtaccTTAATCCACCTCCTCAATGGTTG
Human-HSPA8-F	tggccatggaggcccgaattcATGTCCAAGGGACCTGCAGT
Human-HSPA8-R	gatccccgcggccgcggtaccTTAATCAACCTCTTCAATGGTGG
Pig-HSPA8-F	tggccatggaggcccgaattcGGATGTCTAAGGGACCTGCAGT
Pig-HSPA8-R	gatccccgcggccgcggtaccTTAGTCAACCTCCTCAATGG
M41-S1-RBD(253)-F	tcgagctcaagcttcgaattcTGATGGCTTTGTATGACAGTAGTTCT
M41-S1-RBD(253)-R	ttatctagatccggtggatccTCAATTGTGTAACGTAAAAGTAGTATT
Beaudette-S1-253-F	tcgagctcaagcttcgaattcTGATGGCTTTGTATGACAGTAGTTCT
Beaudette-S1-253-R	ttatctagatccggtggatccTCAATTGTGTAACGTAAAAGTAGTATT
H120-S1-253-F	tcgagctcaagcttcgaattcTGATGGCTTTGTATGACAGTAGTTCT
H120-S1-253-R	ttatctagatccggtggatccTCAAAACCACAAGCCATTATTA
QX-S1-253-F	tcgagctcaagcttcgaattcTGATGAATTTGTTTGATTCTGATAATA
QX-S1-253-R	ttatctagatccggtggatccTCACGCCAGAGTAGTATTAACACTA

*The sequences with lower-case letter are homologous sequences to the selected vectors and the sequences with upper-case letter are the primer sequences for gene amplification.*

### Immunoprecipitation and Co-immunoprecipitation Assays

The membrane proteins from IBV M41 infected CEK cells were extracted using Membrane protein extraction kit (Beyotime) following the manufacturer’s protocol. Membrane protein extracts were immunoprecipitated (IP) with mAbs 1H1 against IBV M41 S protein, as well as mAb against mouse IgG. The precipitated proteins were detected by silver staining and Western blotting using anti-S and anti-HSPA8 antibodies. 293T cells were cotransfected with flag-n/EGFP-C3-M41-RBD, flag-n-HSPA8/EGFP-C3-M41-RBD, flag-n-HSPA8/EGFP-C3-BD-253, flag-n/EGFP-C3-BD-253, flag-n-HSPA8/EGFP-C3-H120-253, flag-n/EGFP-C3-H120-253, flag-n-HSPA8/EGFP-C3-QX-253, flag-n/EGFP-C3-QX-253, flag-human-HSPA8/EGFP-C3-M41-253 and flag-pig-HSPA8/EGFP-C3-M41-253 and then flag-tagged HSPA8 was immunoprecipitated with flag antibody and captured by Sepharose A/G beads. The precipitated proteins were then detected by Western blotting using anti-flag and anti-GFP antibodies. On the other hand, 293T cells were cotransfected with flag-n-HSPA8/EGFP-C3-M41-RBD and flag-n-HSPA8/EGFP-C3 and then GFP-tagged M41-RBD immunoprecipitated with GFP antibody and captured by Sepharose A/G beads. The precipitated proteins were then detected by Western blotting using anti-flag and anti-GFP antibodies.

### Western Blot Analysis

Cell lysates and immunoprecipitated proteins in protein loading buffer were subjected to SDS-PAGE and transferred onto a nitrocellulose membrane. After being blocked with 5% skimmed milk, the membranes were incubated with the indicated primary antibodies at 4°C overnight. After three washes with PBS, the membranes were incubated with HRP-labeled secondary antibody at room temperature for 1 h. The protein bands were then visualized using enhanced chemiluminescence reagent and imaged using AI680 Imager (GE Healthcare).

### Mass Spectrometry

Specific band in silver stained gel was cut and sent for commercial mass spectrometry (LC-MS/MS) analysis carried out by Shanghai Applied Protein Technology Company.

### Indirect Flow Cytometry

CEK, Vero and 293T cells were cultured in two 10 cm dishes and were harvested and washed three times with ice cold PBS. Then, 1 μg/ml of the mAb against HSPA8 (ab19136) was added into the cell suspensions and incubated for 30 min at 4°C. After that, the cells were washed 3 times by repeat centrifugation at 400 g for 5 min and the final pellets were resuspended in ice cold PBS. The fluorochrome-labeled secondary antibody was diluted (1:400) in 3% BSA/PBS and mixed with above cell suspension and again incubated for 30 min at 4°C in the dark. The cells were washed 3 times by centrifugation at 400 g for 5 min and resuspended in ice cold PBS. Finally, samples were analyzed by the flow cytometer (Beckman coulter FC500).

### GST Pull Down Assay

GST, GST-M41-RBD, GST-HSPA8, and His-HSPA8 recombinant proteins were expressed in *Escherichia coli* BL-21 and then purified using glutathione-sepharose beads and Ni-NTA agarose beads according to the manufacturer’s protocol. GST, GST-M41-RBD, and GST-HSPA8 recombinant proteins were bound to glutathione-sepharose beads at 4°C for 4 h. After washing five times with PBS, the beads were incubated for 12 h at 4°C with His-HSPA8 protein and EGFP-C3-M41-RBD cell lysates. After washing the beads five times, the protein complex was dissociated from the beads by boiling with 4xSDS-PAGE loading buffer for 10 min, run on SDS-PAGE, and subjected to Western blot analysis using antibodies against GST tag, 6xHis tag and GFP tag.

### RT-qPCR

Total cellular RNA was isolated by the Trizol reagent (Vazyme) according to the manufacturer’s instructions. One microgram of total RNA was transcribed into cDNA using a reverse transcription kit (Vazyme). The relative abundance of viral RNA and mRNAs was analyzed using the ChamQ Universal SYBR RT-qPCR master mix (Vazyme) and the LightCycler 96 sequence detector system (Roche). Primers 5′-GAAGAAAACCAGTCCCAGA-3′ and 5′-TTACCAGCAACCCACAC-3′ were used to detect IBV viral RNA (Kint et al., 2016), the primers 5′-CATCACAGCCAC ACAGAAG-3′ and 5′-GGTCAGGTCAACAACAGAGA-3′ were used to detect chicken GAPDH mRNA (Kint et al., 2016). The primers 5′-GATCTGGCACCACACCTTCT-3′ and 5′-GGGGTGTTGAAGGTCTCAAA-3′ were used to detect African green monkey β-actin mRNA (Wang et al., 2019). The primers 5′-TGACCAGGGTAACAGGACCA-3′ and 5′-ACGCCCAATCAACCGTTTTG-3′ were used to detect chicken HSPA8 mRNA.

### Binding Blocking Assay

IBV M41 (TCID_50_ = 10^6.5^/ml, 200 μl) was incubated with recombinant protein GST-HSPA8 (100 μg) and control recombinant protein GST (100 μg) at 37°C for 1 h. CEK cells cultured in 6-well plates were incubated with GST-HSPA8-treated virus and GST-treated virus at 4°C for 1 h. After the incubation, the cells were harvested after 3 washes with PBS. Cell-associated viral RNA was quantified by RT-qPCR which indicated the degree of inhibition of virus binding on the host cells caused by recombinant HSPA8. IBV-Beaudette strain (moi = 10) was incubated with recombinant GST-HSPA8 (100 μg) protein and control protein GST (100 μg) at 37°C for 1 h. Before addition of virus to the Vero cells, 400 μl of heparanase I (5 mIU/ml) was added to the cells and incubated at 37°C for 1 h. After washing with PBS, the cells were then incubated with GST-HSPA8-treated virus and GST-treated virus at 4°C for 1 h. After incubation, the cells were harvested after 3 washes with PBS. Cell-associated viral RNA was quantified by RT-qPCR which indicated the inhibition level of recombinant HSPA8 on IBV Beaudette binding to the Vero cells.

### Infection Inhibition Assay

CEK cells were cultured in 6-well plates, and incubated with polyclonal antibody against HSPA8 (2 μg, 2μl/well or 4 μg, 4μl/well) and rabbit-IgG (4 μg, 4 μl/well) at 37°C for 1 h. The cells were then incubated with IBV M41 (TCID_50_ = 10^6^.^5^/ml, 50 μl/well) at 4°C for 1 h, after three washes with PBS, the cells were then transferred to incubate at 37°C for 1 h or 24 h. The cells were harvested 1 hpi or 24 hpi after 3 washes with PBS and subjected to RT-qPCR (1 and 24 hpi) and Western blot analysis (24 hpi). The culture supernatants were also collected 24 hpi for TCID_50_ assay. For inhibition assay on IBV Beaudette strain, Vero cells were cultured in 6-well plates and incubated with 400 μl of heparinase I (5 mIU/ml) at 37°C for 1 h. After 3 washes with PBS, the cells were then incubated with polyclonal antibody against HSPA8 (2 μg, 2 μl/well or 4 μg, 4 μl/well) and rabbit-IgG (4 μg, 4 μl/well) at 37°C for 1 h. The cells then incubated with IBV Beaudette strain (moi = 1) at 4°C for 1 h, after three washes with PBS, the cells were then transferred to incubate at 37°C for 1 or 24 h. Similarly, the cells were harvested 1 or 24 hpi after 3 washes with PBS and subjected to RT-qPCR (1 and 24 hpi) and Western blot analysis (24 hpi). The culture supernatants were also collected 24 hpi for TCID_50_ assay.

### Statistical Analysis

All data are presented as means ± standard deviations (SDs) and analyzed by GraphPad. Significant differences between two groups were analyzed using Student’s *t*-test. *P*-values of differences between means are represented in figures as follows: ^∗∗∗^*P*<0.001; ^∗∗^*P*<0.01; ^∗^*P*<0.05; and ns (nonsignificant), *P* > 0.05.

## Results

### HSPA8 Was Identified as Cell Membrane Protein That Interacts With Spike Protein of IBV

**FIGURE 1:**
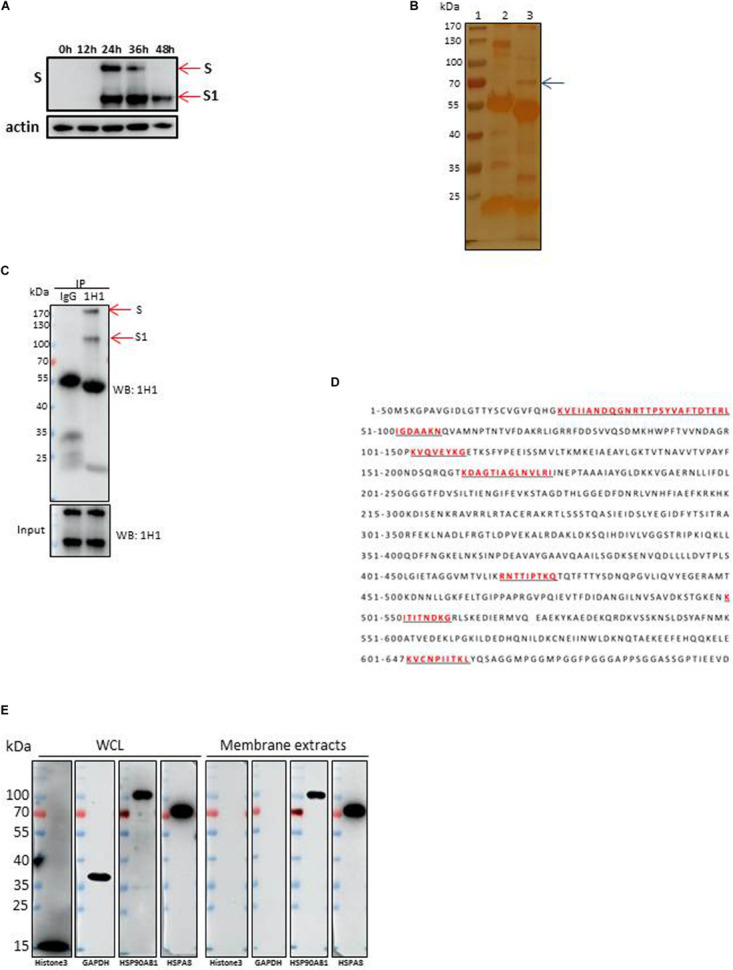
Identification of HSPA8 as a target membrane molecule for IBV Spike protein. **(A)** Determination of viral protein expression level of IBV M41 strain in CEK cells. CEK cells were infected with IBV M41 (TCID_50_ = 10^6.5^/ml, 50 μl/well) in 6-well plates. Infected cells were harvested at 0, 12, 24, 36, and 48 h post-infection. The whole cell lysates were subjected to SDS-PAGE and Western blot analysis with mAb 1H1 to spike protein. The expression of cellular actin protein was used as control. **(B)** Silver staining of membrane proteins of CEK cells after immunoprecipitation assay. Membrane protein extracts of IBV M41 infected CEK cells were immunoprecipitated with mAb 1H1 (lane 3) to IBV M41 spike protein, and mouse IgG (lane 2). Lane 1, protein marker. Arrow indicated the protein band at 70kDa specifically precipitated by mAb 1H1. **(C)** Western blot analysis of membrane proteins of CEK cells after immunoprecipitation assay. Sample orders were same as shown in **(B)**. **(D)** Amino acid sequence of HSPA8. The gel fragment indicated with the arrowhead in **(B)** was analyzed by LC-MS/MS. The resulting peptide sequences of HSPA8 were underlined and marked as red color. Full length HSPA8 sequence was searched against chicken protein database in Uniprot. **(E)** Western blot analysis of HSPA8 present in the membrane protein extracted from CEK cells. Membrane protein fraction of CEK cells and whole cell lysates of CEK cells were immunoblotted with antibodies against the cytoplasmic protein marker (GAPDH), nuclear protein marker (Histone3), membrane protein marker (HSP90AB1) and HSPA8. WCL, whole cell lysates.

Up to now, little is known about which membrane proteins of CEK cells could interact with IBV spike protein and facilitate the infection by IBV. To address this question, the IBV-M41 strain was passaged in CEK cells to get a CEK culture adapted IBV M41 virus strain. As showed by Western blot analysis of the viral proteins, the expression level of S1 protein of IBV M41 reached a peak at 36 h post-infection (Figure 1A), which was selected as the optimal time point for preparation of immunoprecipitated S protein in the following experiments. Western blot analysis showed that the membrane proteins could be efficiently extracted with membrane protein extraction kit as the extracted proteins could react with antibody to membrane protein marker HSP90AB1 but not with antibodies to a cytoplasmic protein (GAPDH) and a nuclear protein (Histone3) (Figure 1E).

To identify membrane proteins that may interact with IBV spike protein, immunoprecipitation assay was performed with membrane proteins from IBV M41-CEK infected cells using mAb 1H1 to IBV spike protein and mouse IgG. Immunoprecipated proteins were resolved by SDS-PAGE before silver staining (Figure 1B). A specific protein band with molecular weight of 70 kDa (arrow) was visualized by silver staining in 1H1 immunoprecipitates from infected cells (Figure 1B, lane 3), but not from uninfected cells (not shown). IBV S protein around 170 kDa and its S1 subunit around 100 kDa were detected by Western blot analysis with mAb 1H1 to IBV M41 S1 (Figure 1C, red arrows). The 70 kDa specific protein band (Figure 1B, lane 3, arrow) which was pulled down by mAb to S1 (1H1) but not by the control antibody was excised and sent for LC-MS/MS analysis. The MS result revealed that the specific 70 kDa band was heat shock protein member 8 (HSPA8), also known as heat shock protein 71. The full length sequence of putative chicken HSPA8 is 647 amino acid in size (Figure 1D). Chicken HSPA8 could be detected from whole cell lysates and membrane extracts of CEK cells (Figure 1E).

### HSPA8 Was Further Confirmed to Be Expressed on the Surface of Host Cells and in Chicken Tissues

**FIGURE 2:**
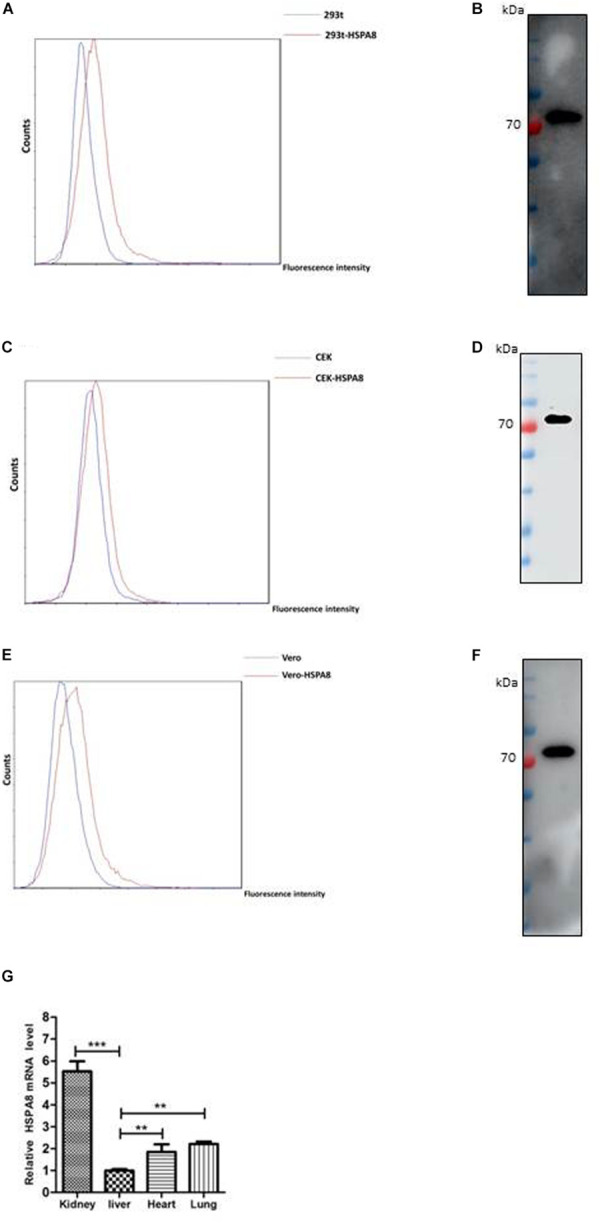
Detection of expression of HSPA8 on cell surface and chicken tissues. **(A,C,E)** Identification of HSPA8 expression on surface of 293T cells **(A)**, CEK cells **(C)** and Vero cells **(E)** by flow cytometry analysis with antibody against HSPA8. Total 6.5 × 10^4^ of 293T cells, CEK cells and Vero cells were used for each analysis. The blue lines mean without primary antibody and the red lines indicated the reaction with HSPA8 antibody. **(B,D,F)** Western blot analysis of the membrane protein extracts of 293T cells **(B)**, CEK cells **(D)** and Vero cells **(F)** with antibody against HSPA8. **(G)** Detection of HSPA8 expression level in 1-day-old chicken tissues by RT-qPCR. Tissue samples collected from three chickens were used for the analysis of HSPA8 expression level. **P < 0.01; ***P < 0.001.

To verify whether HSPA8 is expressed on the cell surface, we performed indirect flow cytometry assay without cell permeabilization. As results shown in Figures 2A,C,E, HSPA8 was found to be expressed on the surface of 293T, Vero and CEK cells. Additionally, the expression was also confirmed by Western blot analysis with mAb to HSPA8 (Figures 2B,D,F).

Furthermore, we checked the expression of HSPA8 in chicken tissues by RT-qPCR. The results showed that HSPA8 was found to be expressed in the tissues of kidney, liver, heart and lung of 1-day-old chickens with highest expression level in kidneys (Figure 2G).

### HSPA8 Interacts With RBD of IBV Spike Protein

**FIGURE 3:**
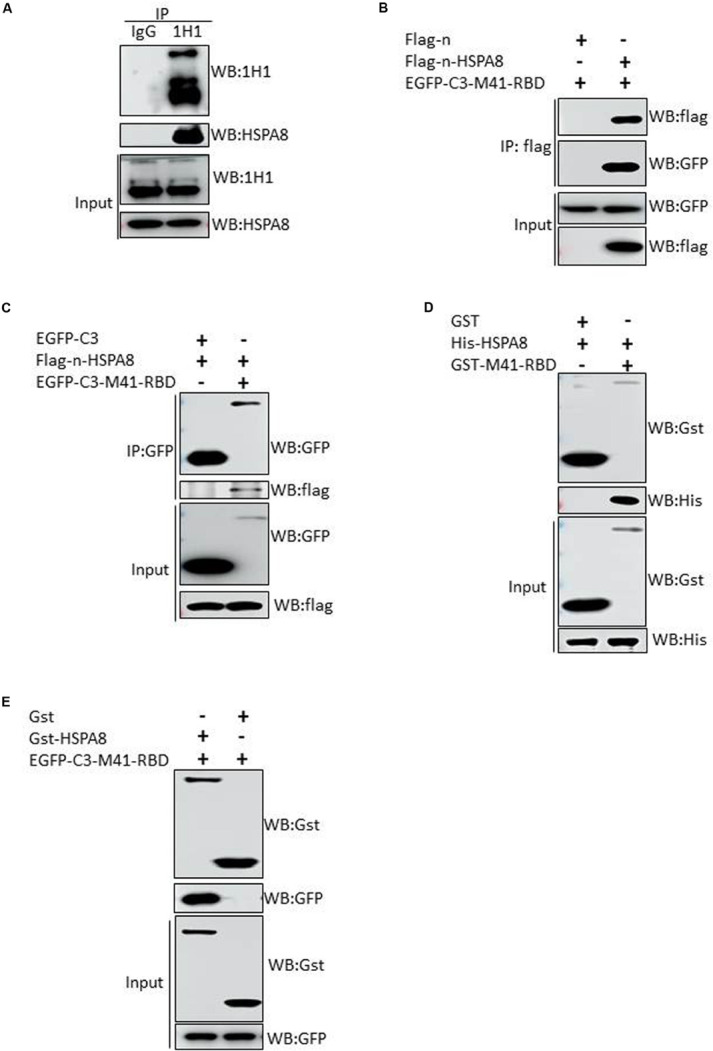
Interaction of HSPA8 with RBD of IBV spike protein. **(A)** Detection of the interaction between HSPA8 and IBV M41 spike protein in M41-infected CEK cells by immunoprecipitation assay using mAb 1H1 to IBV S and mouse IgG. IBV S1 and HSPA8 in precipitates were detected by immunoblots with mAbs to IBV S1 and HSPA8. **(B)** Detection of the interaction between HSPA8 and IBV M41-RBD by co-IP assay using HSPA8 as bait protein. 293T cells were cotransfected with indicated plasmids and then flag-tagged HSPA8 was immunoprecipitated with anti-flag antibody. The precipitated proteins were detected by Western blotting using anti-flag and anti-GFP antibodies. **(C)** Detection of the interaction between HSPA8 and IBV M41-RBD by reciprocal co-IP assay using M41-RBD as bait protein. 293T cells were cotransfected with indicated plasmids and then GFP-tagged M41-RBD was immunoprecipitated with anti-GFP antibody. The precipitated proteins were detected by Western blotting using anti-flag and anti-GFP antibodies. **(D,E)** Identification of the direct interaction between HSPA8 and IBV M41-RBD by GST-pull-down assay. In **(D)**, GST and GST-M41-RBD recombinant proteins were separately bound to glutathione-sepharose beads and then incubated with His-HSPA8 recombinant protein. The protein complexes were detected by Western blot analysis with antibodies against GST and 6xHis. In **(E)**, The lysates of 293T cells transfected with EGFP-C3-M41-RBD vector were incubated with GST or GST-HSPA8 protein which was separately bound to glutathione-sepharose beads. Subsequently, the protein complexes were detected by Western blot analysis with antibodies against GST and GFP.

To further verify the interaction between IBV M41 spike protein and HSPA8 of host cell, we performed immunoprecipitation assay with mAb 1H1 to spike protein in M41 infected CEK cells. As shown in Figure 3A, HSPA8 could be immunoprecipated with IBV spike protein, but not with mouse IgG. These results indicated that HSPA8 could interact with IBV spike protein. To examine whether HSPA8 interacts with spike protein through the RBD, we performed co-IP assay with lysates of transfected cells. As shown in Figure 3B, M41-RBD specifically immunoprecipitated with HSPA8, but not with the empty vector control. To further confirm the interaction between HSPA8 and M41-RBD, reciprocal co-IP was performed using M41-RBD as bait protein. The results showed that HSPA8 could be immunoprecipitated with M41-RBD, but not with the empty vector control (Figure 3C).

Next, *in vitro* GST-pull-down assay was also performed to further confirm the direct interaction between HSPA8 and M41-RBD. The results indicated that HSPA8 could specifically interact with M41-RBD, but not with the control GST protein (Figures 3D,E).

### Recombinant HSPA8 Protein and Anti-HSPA8 Antibody Block/Inhibit IBV Infection of CEK Cells

**FIGURE 4:**
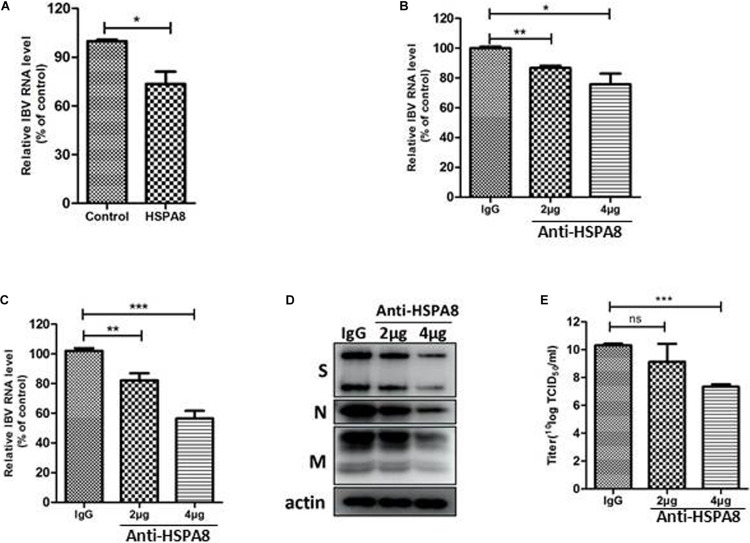
Inhibition of IBV M41 binding to CEK cells by binding blocking assay and infection inhibition assay. **(A)** Recombinant HSPA8 blocked the binding of IBV M41 to CEK cells. IBV M41 (TCID_50_ = 10^6.5^/ml, 200 μl) was incubated with recombinant protein GST-HSPA8 (100 μg) and control recombinant protein GST (100 μg) at 37°C for 1 h. CEK cells cultured in 6-well plates was incubated with GST-HSPA8-treated virus and GST-treated virus at 4°C for 1 h. After 1 h incubation, the cells were harvested after 3 washes with PBS. The viral RNA associated with cells was determined by RT-qPCR. **(B–E)** Anti HSPA8 antibody inhibited IBV infection in CEK cells. CEK cells were cultured in 6-well plates and incubated with polyclonal antibody against HSPA8 (2 μg, 2 μl/well or 4 μg, 4 μl/well) and rabbit-IgG (4 μg, 4 μl/well) at 37°C for 1 h. The cells were then incubated with IBV M41 virus (TCID_50_ = 10^6.5^/ml, 50 μl/well) at 4°C for 1 h then incubated at 37°C. Cells were harvested after 3 washes with PBS at 1 hpi **(B)** and 24 hpi **(C,D)**. The inhibition level was determined by RT-qPCR **(B,C)** and Western-blotting **(D).** Virus titer in culture supernatant at 24 hpi was determined by TCID_50_ assay **(E)**. Data shown in this figure represent those from three independent experiments. *P < 0.05; **P < 0.01; ***P < 0.001; ns (nonsignificant), *P* > 0.05.

Binding blocking assay was performed using recombinant HSPA8 as competitor to confirm its role in IBV infection. As shown in Figure 4A, the amount of viral RNA associated with cells following incubation at 4°C with virus pre-incubated with HSPA8 was less than the amount of viral RNA associated with cells incubated with virus pre-incubated with GST protein.

In addition, an inhibition assay was also performed to check whether antibody to HSPA8 could inhibit IBV M41 infection of CEK cells. As shown in Figures 4B,C, infection of IBV was significantly reduced in anti HSPA8 polyclonal antibody pretreated cells at both 1 hpi and 24 hpi. A similar result was also observed in Western blot analysis at 24hpi (Figure 4D). Virus titer in infected cell culture supernatant at 24 hpi from the anti HSPA8 polyclonal antibody pretreated cells was lower than in that from control antibody pretreated cells (Figure 4E). Previous studies have shown that expression of functional cellular receptor in nonpermissive cells could make the cell susceptible to virus infection (Zhang et al., 2018; Wang and Huang, 2019). However, IBV M41 could not be passaged in an HSPA8-overexpressing cell line (Supplementary Figure 1).

### HSPA8 Interacts With the N-Terminal 19–272 Amino Acids of S1 of Other IBV Strains and M41-RBD Interacts With HSPA8 of Human and Pig

**FIGURE 5:**
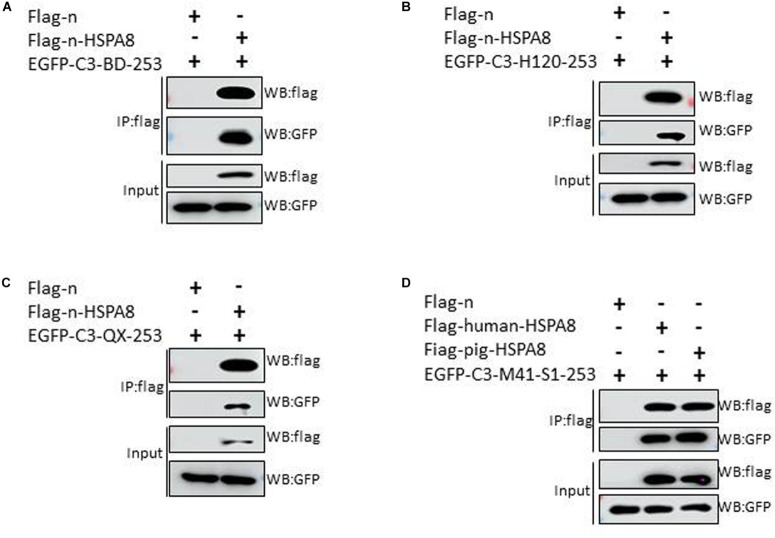
HSPA8 interacts with the N-terminal 19–272 amino acids of S1 subunit of IBV Beaudette, H120 and QX strains and M41-RBD interacts with HSPA8 of human and pig. **(A–C)** Detection of interaction between chicken HSPA8 and the N-terminal 19–272 amino acids of the S1 subunit of Beaudette **(A)**, H120 **(B),** and QX **(C)** strains. 293T cells were cotransfected with indicated plasmids and then flag-tagged HSPA8 was immunoprecipitated with anti-flag antibody. The precipitated proteins were detected by Western blotting using anti-flag and anti-GFP antibodies. **(D)** Detection of the interaction between IBV M41-RBD and HSPA8 of human and pig. 293T cells were cotransfected with indicated plasmids and then flag-tagged HSPA8 was immunoprecipitated with anti-flag antibody. The precipitated proteins were detected by Western blotting using anti-flag and anti-GFP antibodies.

Apart from the M41-RBD, we also performed similar experiments with the N-terminal 19–272 amino acids of the S1 subunit of other IBV strains. The results showed that the HSPA8 could interact with the N-terminal 19–272 amino acids of S1 of IBV Beaudette, H120 and QX strains (Figures 5A–C). Surprisingly, HSPA8 from human and pig also interacted with M41-RBD as indicated by co-IP assay (Figure 5D).

### HSPA8 Plays a Role in IBV Beaudette Infection of Vero Cells

**FIGURE 6:**
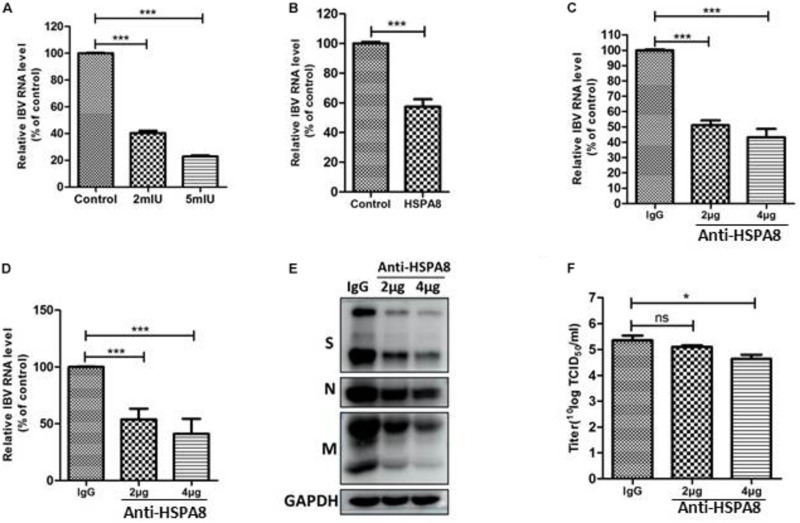
Inhibition of IBV Beaudette infection in Vero cells by binding blocking assay and infection inhibition assay. **(A)** Confirmation of the effects of removal of HS on IBV Beaudette infection. Before infection, 400 μl of heparinase I at different concentration (0, 2, 5 mIU/ml) was added to Vero cells at 37°C for 1 h. The cells were then incubated with IBV Beaudette strain (moi = 1) at 4°C for 1 h. After incubation, the cells were harvested after 3 washes with PBS. The viral RNA associated with cells was determined by RT-qPCR. **(B)** Recombinant HSPA8 blocked the binding of IBV Beaudette to Vero cells. IBV Beaudette (moi = 10) was incubated with recombinant protein GST-HSPA8 (100 μg) or control protein GST (100 μg) at 37°C for 1 h and then added to Vero cells pretreated with 400 μl of heparanase I (5 mIU/ml) and incubated at 4°C for 1 h. The cells were harvested after 3 washes with PBS. The viral RNA associated with cells was quantified by RT-qPCR. **(C–F)** Anti-HSPA8 antibody inhibited IBV-Beaudette infection of Vero cells. Vero cells pretreated with 400 μl of heparanase I (5 mIU/ml) were incubated with polyclonal antibody against HSPA8 (2 μg, 2 μl/well or 4 μg, 4 μl/well) and rabbit-IgG (4 μg, 4 μl/well) at 37°C for 1 h. The cells were then incubated with IBV Beaudette strain (moi = 1) at 4°C for 1 h then incubated at 37°C. Cells were harvested after 3 washes with PBS at 1 hpi **(C)** and 24 hpi **(C,D)**. The inhibition level was quantified by RT-qPCR **(C,D)** and Western-blotting **(E).** Virus titer in culture supernatant at 24 hpi was determined by TCID_50_ assay **(F)**. Data shown in this figure represent those from three independent experiments. *P < 0.05; **P < 0.01; ***P < 0.001; ns (nonsignificant), *P* > 0.05.

IBV-Beaudette strain could bind to heparan sulfate (HS) through its HS binding site in S2 subunit (Madu et al., 2007). To exclude the influence of HS on the surface of Vero cells for IBV Beaudette binding, heparanase I was used to remove HS on the surface of Vero cells. As shown in Figure 6A, the viral RNA associated with cells was significantly reduced in heparanase I-pretreated cells compared to the control cells. These results indicated that pretreatment with heparanase I could inhibit IBV Beaudette binding to Vero cells. Further, the importance of HSPA8 in IBV-Beaudette strain infection was also studied by binding blocking assay and infection inhibition assay after removal of HS with heparanase I. As shown in Figure 6B, when HS was removed, recombinant HSPA8 was able to block IBV Beaudette strain binding to Vero cells. In the case of infection inhibition assay, the amount of viral RNA was significantly reduced in HSPA8 antibody pretreated cells compared to the control IgG pretreated cells (Figures 6C,D). Similar result was observed in Western blot analysis which showed that infection of IBV was significantly inhibited by HSPA8 antibody compared to the control IgG (Figure 6E). Virus titer in infected cell culture supernatant from the anti HSPA8 antibody pretreated cells was also lower than control antibody pretreated cells (Figure 6F).

## Discussion

In this study, we performed immunoprecipitation assay with membrane proteins from IBV M41 infected CEK cells and found that HSPA8 was involved in the interaction of host cell membrane and IBV spike protein (Figure 1). Then, we confirmed the expression of HSPA8 on the plasma membrane of different cell types by indirect flow cytometry assay (Figures 2A,C,E), and by Western blot analysis (Figures 2B,D,F). The result of immunoprecipitation and prevalence of HSPA8 on host cell membrane suggested its putative role during initial stages of virus infection. Interestingly, the expression level of HSPA8 in kidney and lung tissues of chicken is higher compared to other organs (Figure 2G). These results suggest that HSPA8 might be involved in tissue tropism of IBV infection in chicken, considering that IBV infection usually causes respiratory distress and kidney damage.

RBD of coronavirus S1 protein is primarily responsible for virus interaction with host cells. The N-terminal 19–272 amino acids of the S1 subunit of IBV-M41 was identified as a RBD (Promkuntod et al., 2014). We found that HSPA8 directly interacted with the RBD of IBV M41-spike protein as confirmed by GST-pull-down assay (Figure 3). Furthermore, HSPA8 also interacted with the RBD-associated N-terminal 19–272 amino acids region of the S1 subunit of IBV Beaudette, H120 and QX strains (Figure 5). Binding blocking assay and infection inhibition assay showed that recombinant HSPA8 protein and antibody against HSPA8 could inhibit the infection of IBV M41 and Beaudette strains in CEK and Vero cells, respectively (Figures 4, 6). These results indicate that HSPA8 acts as a target molecule on host cell membrane for IBV spike protein and is involved in IBV infection.

However, HSPA8 overexpression in the nonpermissive cells did not facilitate IBV infection (Supplementary Figure 1) and the HSPA8 antibody did not completely abolish the infection of IBV of the host cells. Considering that many binding molecules on the cell surface are involved in coronavirus infection using different strategies, we hypothesize that the HSPA8 might act as an attachment factor during the IBV infection.

It has been widely known that HSPA8 has multiple functions including as a receptor for virus infection (Sagara et al., 1998; Guerrero and Moreno, 2012). In this study, for the first time, HSPA8 was found to play a role in IBV infection. Zhang et al. (2017) identified HSP70 protein, one of the members of HSP70 family, as a part of the receptor complex for IBV. HSP70, which acts as a receptor associated protein, is different from previously identified carbohydrate like receptors such as α-2, 3 sialic acid, and HS, suggesting that IBV could exploit both protein and carbohydrate molecules alike to gain entry into the target cells. HSPA8 and HSP70 both belong to the HSP70 family and their amino acid sequences share 86% identity. Thus, it is quite possible that HSPA8 exhibits similar function as HSP70 during IBV infection.

HS has been reported to co-localize with heat shock protein (HSP) 90 (Snigireva et al., 2015). To check whether HS on cell membrane may have influenced IBV S protein binding to HSPA8, we performed the binding blocking assay and infection inhibition assay based on removal of HS by using heparanase. The results showed that the recombinant HSPA8 and antibody to HSPA8 could still block/inhibit IBV infection of host cells, suggesting that HSPA8 plays a role during IBV infection independent from HS (Figures 6B–F).

In this study, we found that unglycosylated RBD which was expressed in *E. coli* could interact with HSPA8. Recent studies showed that the glycosylation of IBV M41-RBD is essential for its binding to chicken tracheal tissue and the removal of the glycosylation modification site of RBD may affect IBV binding to host cells (Parsons et al., 2019; Bouwman et al., 2020). Considering that HSPA8 is not a functional receptor for IBV and the importance of glycosylation of RBD for binding to host cells, we speculate that unglycosylated RBD might not act like glycosylated RBD to interact with more host molecules. On the other hand, IBV S1 RBD tends to bind sialylated glycan receptor on the surface of host cells (Bouwman et al., 2020), and it has been reported that HSPA8 could also be glycosylated (Guinez et al., 2010), therefore the glycosylation of HSPA8 may increase the possibility for IBV binding to the host cells.

Finally, in this study, HSPA8 from human and pig was also found to interact with M41-RBD (Figure 5D), and sequence analysis showed that the similarity of amino acid sequence among HSPA8 of human, pig and chicken was more than 96%, indicating that the role of HSPA8 on coronavirus binding might be conserved among different host species.

Taken together, our results suggest that HSPA8 plays an important role during initial stage of IBV infection. The identification of HSPA8 as an attachment factor for IBV infection will increase our understanding of the mechanism of IBV pathogenesis.

## Data Availability Statement

All datasets generated for this study are included in the article/Supplementary Material.

## Ethics Statement

The animal study was reviewed and approved by the Committee on the Ethics of Animal of Zhejiang University (ZJU20170388).

## Author Contributions

ML, JZ, and PZ designed the experiments. PZ, CL, and CF performed the experiments. PZ, ML, and JZ analyzed the data. HS, PX, and YY prepared the reagents. PZ, ML, and NK drafted the manuscript. All authors read and approved the final manuscript.

## Conflict of Interest

The authors declare that the research was conducted in the absence of any commercial or financial relationships that could be construed as a potential conflict of interest.
