# Supplementary material for: Heat Shock Protein Member 8 Is an Attachment Factor for Infectious Bronchitis Virus
Source: Front Microbiol. 2020 Jul 10;11:1630. doi: 10.3389/fmicb.2020.01630 (PMC7381282; doi:10.3389/fmicb.2020.01630)
Supplement: Supplementary file 1 [file Data_Sheet_1.PDF]

Supplementary Material

METHODS

Construction of HSPA8-overexpressing-cell line

HSPA8 gene fragment with a flag tag fused at the C-terminus was obtained by two step PCR with the primers listed in Table 2 and then cloned into the lentiviral PCDH-CMV-MCS-EF1 vector between restriction enzyme *Eco*RI and *Bam*HI sites by homologous recombination, resulting in a positive vector PCDH-CMV-MCS-EF1-HSPA8-flag. 293T cells were cotransfected with PCDH-CMV-MCS-EF1-HSPA8-flag/PMD/PSPAX2.0 vectors with ratio 4:2:2. Thirty-six hours post transfection, the supernatant of transfected 293T cells was collected and used to infect Vero cells, which were then selected by puromycin to obtain an HSPA8-overexpressing-cell line.

TABLE 2 | Primers used for amplified gene fragments

**Primers**	**Sequence (5'-3')**
PCDH-CMV-MCS-EF1-HSPA8-ECORI-F	gattctagagctagcgaattcATGTCAAAGGGACCAGC
HSPA8-flag-end-R	TCACTTGTCATCGTCGTCCTTGTAGTCCATATCCACCTCCTCAATGGT
PCDH-CMV-MCS-EF1-BamHI-flag-R	atccttcgcggccgcggatccTCACTTGTCATCGTCGTC

Note: The sequences with lower-case letter are homologous sequences to the selected vectors and the sequences with upper-case letter are the primer sequences for gene amplification.

Supplementary Figures

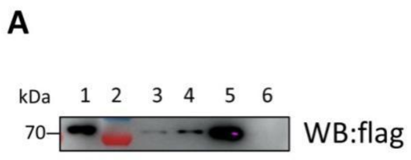

B

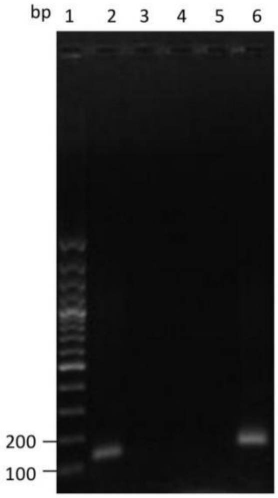

Supplementary Figure 1. (A) Detection of the expression of HSPA8-flag in HSPA8-overexpressing-cells by Western blot analysis. Anti-flag antibody was used to detect the expression of HSPA8-flag. lane 1, positive control; lane 2, protein marker; lane 3-lane 5, first to third subcultures of the HSPA8-overexpressing-cell line; lane 6, negative control. **(B)** Detection of blind passages of IBV M41 in HSPA8-overexpressing-cell by RT-PCR. lane 1, Nucleic acid marker; lane 2-lane 4, the first to third passages of IBV M41-infected HSPA8-overexpressing-cells; lane 5, negative control; lane 6, positive control.
